# Supplementary material for: Coral cover surveys corroborate predictions on reef adaptive potential to thermal stress
Source: Sci Rep. 2020 Nov 12;10:19680. doi: 10.1038/s41598-020-76604-2 (PMC7661510; doi:10.1038/s41598-020-76604-2)

Supplementary information for

Coral cover surveys corroborate predictions on reef adaptive potential to thermal stress

Oliver Selmoni, Gaël Lecellier, Laurent Vigliola, Véronique Berteaux-Lecellier, Stéphane Joost

Supplementary Figure 1. Predictive accuracy evaluation of genotype-environment association models. For every potentially adaptive genotype against heat uncovered in a previous seascape genomics study (Selmoni et al., 2020), the resulting association model was cross-validated using a "leave-one-population-out" approach. The plots display the correlation between the expected and observed genotype frequencies. In the three boxplots on the left, the predictive accuracy is evaluated for potentially adaptive genotype-environment association models of the three studied species (*Acropora millepora, Pocillopora damicornis and Pocillopora acuta*). The three boxplots on the right display the results of the same cross-validation approach on 1000 random genotypes from the genome of the three species.

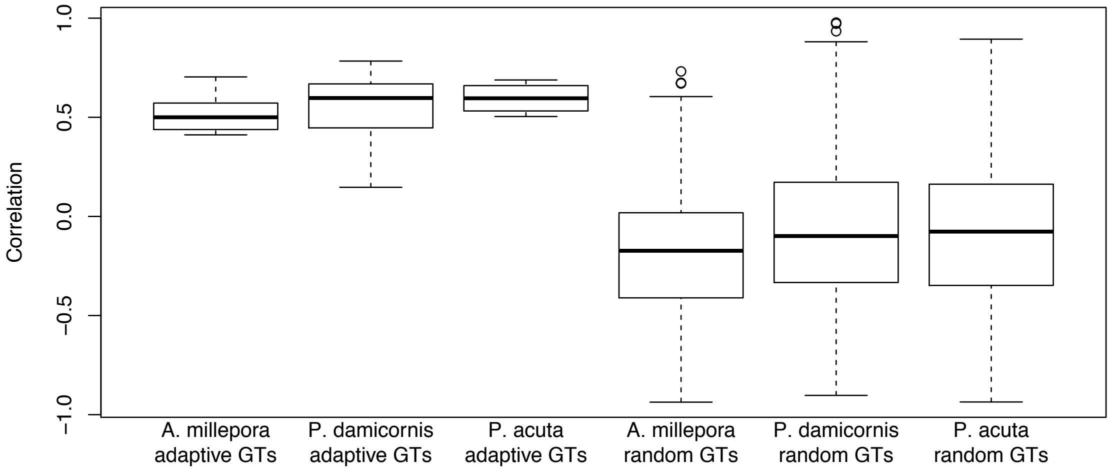

Supplementary Figure 2. Observed frequency of adaptive genotypes to heat. The three boxplots display, for each of the three studied species (a: *Acropora millepora*, b: *Pocillopora damicornis*, c: *Pocillopora acuta*), the observed frequency of genotypes putatively adaptive to heat stress (y-axis) at reefs predicted with different values of probability of heat stress adaptation (low PA:PA<0.6, high PA: PA≥0.6) and inbound connectivity index (low ICI: ICI<1500 km^2, high ICI: ICI≥1500 km^2).

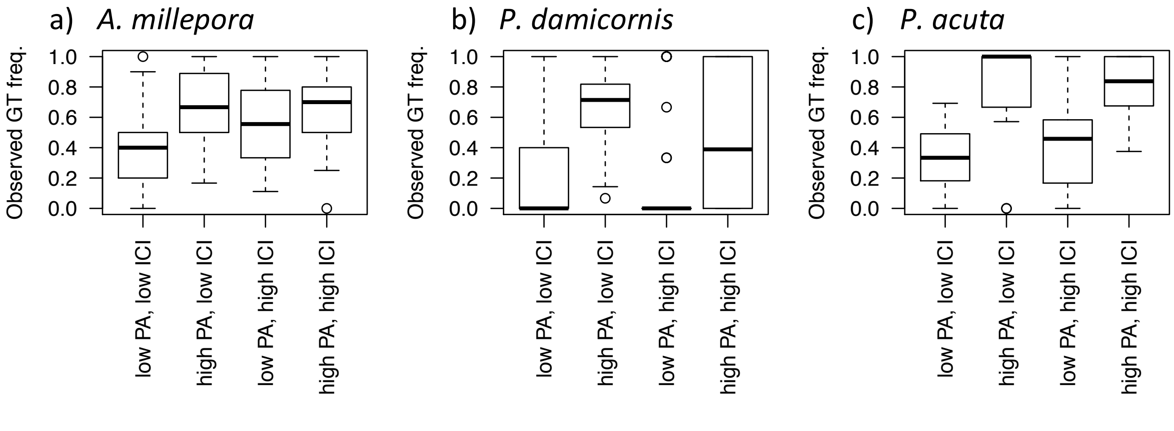

Supplementary Figure 3. Boundaries of the study area. The purple area displays the boundaries of the study area, located approximately 250 km around the most peripheral reefs of New Caledonia (excluding Chesterfield Reefs, located in the western part of the archipelago). Map prepared using R (v. 3.5; R Core Team, 2016).

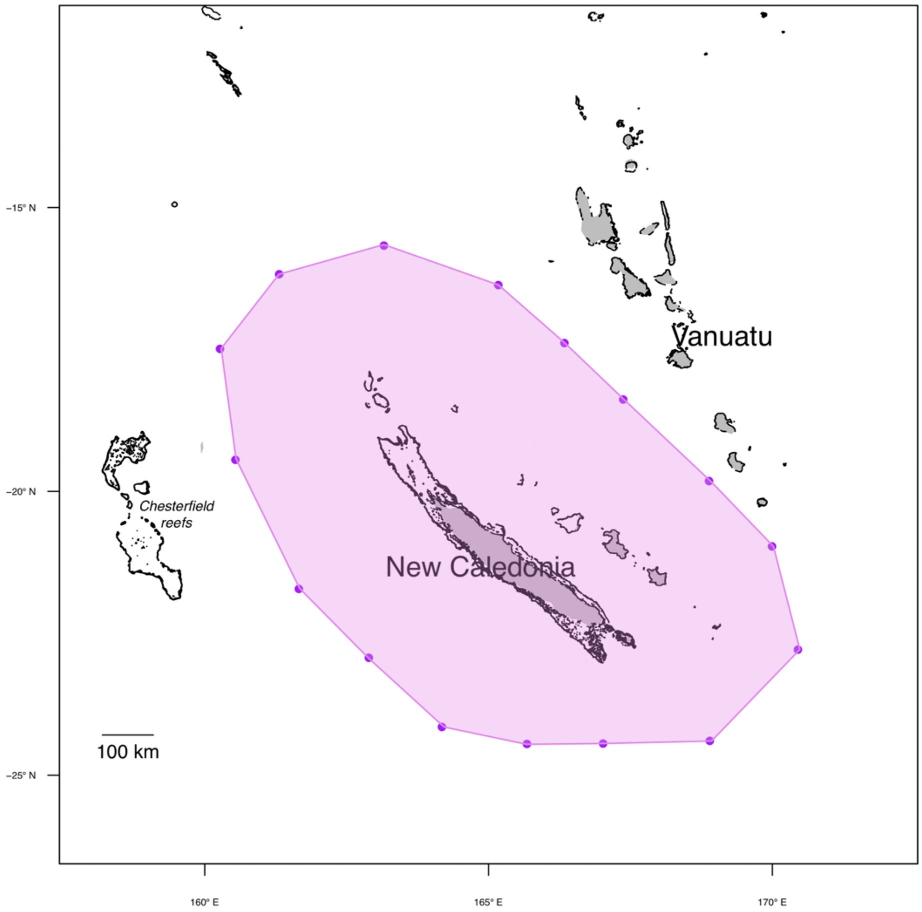

Supplement: Supplementary file 1 — Supplementary information. [file 41598_2020_76604_MOESM1_ESM.pdf]
